# Relationship between the Toothbrushing Behavior and Hand Hygiene Practices of Korean Adolescents: A Study Focused on the 15th Korea Youth Risk Behavior Survey Conducted in 2019

**DOI:** 10.3390/ijerph18115913

**Published:** 2021-05-31

**Authors:** Eun-Jeong Kim, Hye-Ju Lee

**Affiliations:** 1 Department of Dental hygiene, Gangdong University, Eumseong-gun 27600, Chungcheongbuk-do, Korea; kej1007@gangdong.ac.kr; 2 Dental Research Institute, School of Dentistry, Seoul National University, Seoul 03080, Gwanak-gu, Korea; 3 Department of Dental Hygiene, College of Health Science, Sun Moon University, Asan-si 31460, Chungcheongnam-do, Korea

**Keywords:** oral hygiene, hand hygiene, toothbrushing, handwashing, adolescent, KYRBS

## Abstract

Hand hygiene is one of the most important measures available to prevent infectious diseases such as COVID-19, and it is recommended that individuals wash their hands periodically before and after meals, after using toilets, before preparing food at home, at schools, and in public places. The aim of this study was to investigate the relationship between oral hygiene and hand hygiene in Korean adolescents. Data from 57,303 adolescents who participated in the 2019 Korea Youth Risk Behavior web-based survey were analyzed to determine the relationship between oral hygiene and hand hygiene. A complex sample logistic regression analysis was performed to determine association between toothbrushing behavior and handwashing practices. According to the results of this study, adolescents who brushed their teeth after lunch were 1.48 times more likely to practice handwashing before lunch than were those who did not brush their teeth after lunch (*p* < 0.001). In addition, the odds ratios adjusted for gender, grade, school type, and residence were found to be 1.87 (*p* < 0.001). Moreover, these adjusted odds ratios were higher in students who received personal hygiene education (OR: 1.98, *p* < 0.001). Oral hygiene practices were found to be related to personal hygiene, as assessed by handwashing, in Korean adolescents. Additional studies are needed to develop ways of improving the hygiene and health of adolescents.

## 1. Introduction

At the end of 2019, as the acute respiratory disease known as COVID-19 spread worldwide, the World Health Organization (WHO) issued guidelines for the prevention and control of infectious diseases. According to the guidelines, hand hygiene is one of the most important measures available to prevent infectious diseases such as COVID-19, and it is recommended that individuals wash their hands periodically before and after meals, after using toilets, before preparing food at home, at schools, and in public places [1]. Other prior studies have also reported that handwashing can prevent a number of infectious diseases, such as SARS, food poisoning, the common cold, influenza, and epidemic eye diseases; moreover, handwashing alone using regular soap can reduce the incidence of pneumonia, bacterial dysentery, and diarrhea by 40–50% [2]. Thus, handwashing is the easiest way to prevent germs and viruses from spreading.

According to a study conducted on handwashing among adolescents in Korea, 13,153 (18.5%) adolescents do not wash their hands before meals at school, indicating that continuous monitoring is necessary [3]. In addition, research on annual handwashing practice rates showed that handwashing practices were significantly higher in 2009, likely due to the prevalence of the swine flu and the consequent effects of hygiene education [4]. Therefore, properly informing adolescents about health concepts and hygiene habits can have a large impact on the prevention of infectious diseases and can serve as a foundation for the promotion of health in adulthood; hence, continuous monitoring is necessary.

Adolescence is a very important period in which permanent dentition develops through mixed dentition. Awareness of the importance of good oral health practices during this period and efforts to promote oral health in adulthood are important factors in maintaining oral health [5]. It has also been reported that oral hygiene practices in adolescence are one of the most important ways to control and prevent oral diseases [6,7]. According to a study on the relationship between oral health practices and oral disease symptoms in Korean adolescents, adolescents with severe oral disease symptoms and poor oral health practices were reported to be at risk for developing poor oral hygiene and poor oral health practices [8]. Another study found that 15.7% of female students aged 12 to 18 years old who brushed their teeth more than three times a day and 73.9% of those who did not brush their teeth at all developed periodontal diseases [9]. However, the average toothbrushing practice rate after lunch, a leading indicator of oral health practices, was 36.9% among adolescents, whereas it was 25.4% for middle-school students and 52.2% for high-school students, and problems with oral health practices in adolescents were reported [10].

To date, several studies related to oral hygiene and personal hygiene in adolescence have been conducted, but most of the studies were cross-sectional studies that reported the statuses at one time point, whereas studies on their associations are lacking. Therefore, the purpose of this study was to provide basic information important for establishing health education in schools and preparing health promotion measures by determining the association between toothbrushing behavior and handwashing practices and to evaluate the impact of personal hygiene education.

## 2. Materials and Methods

### 2.1. Study Subjects and Study Design

This study was conducted by using raw data from the 15th Korea Youth Risk Behavior survey conducted in 2019 [11]. This survey is an online anonymous survey that does not collect any personal information of the participants, and it is a nationally approved statistical survey (No. 117058) that has been approved by the state in accordance with Article 18 of the Statistical Act of the Republic of Korea, not approved by the institutional ethics committee. All students who participated in the survey participated in the survey after informed consent. A total of 57,303 middle- and high-school students from a total of 800 schools nationwide participated in the survey. The extraction frames at the sampling stage were used to retrieve data from middle and high schools nationwide in April 2018, and the high schools were divided into general and specialized high schools on the basis of the classification of the extraction frames. In the population stratification phase, to minimize sampling error, 39 local groups and school levels were used as stratification variables to divide the population into 117 layers. Local provinces were classified into 17 metropolitan, small, and medium cities, and 39 cities were classified by geographical accessibility, the number of schools, population size, the living condition, the smoking rate, and the alcohol drinking rate. In the sample allocation phase, the sample size was 400 middle schools and 400 high schools, and five middle and high schools were selected from each of the 17 cities and provinces. The proportional allocation method was used to match the study population and overall population compositions by the variables used for stratification, and the number of sample schools was determined according to city and province (large city, small and medium city, county area), local province, middle school (male and female), engineering high school (male and female), general high school, and specialized high school. The sampling method was used for stratified colony extraction, with the primary extraction unit being school and the secondary extraction unit being class. For the first extraction, sample schools were selected using the permanent random number extraction method for each strata. The secondary extraction was performed at random from the selected sample schools with one class per grade [11].

### 2.2. Survey Methods

The variables used in this study consisted of a total of six: four general characteristics of the study subjects, toothbrushing practice, and handwashing practice. The general characteristics that were investigated included gender, school type (coeducational, all-boys, and all-girls schools), grade year and type of residence (metropolis, city, and rural area). The dependent variable was handwashing before meals at school (yes/no), and the independent variable was toothbrushing after lunch (yes/no). Moreover, we investigated whether students had ever received personal hygiene education at school within the last 12 months.

### 2.3. Statistical Analysis

A frequency analysis of the complex samples was conducted to assess the distributions of the demographic and sociological characteristics of the study subjects. Chi-square tests of complex sample analysis were conducted to determine whether individuals used a toothbrush after lunch and whether they washed their hands before a meal at school. A complex sample logistic regression analysis was performed to determine whether there was an association between handwashing practices and toothbrushing practices. This analysis was also performed by personal hygiene education. The data were analyzed using SPSS 19.0 (IBM Co., Armonk, NY, USA), and the significance level was 0.05.

## 3. Results

### 3.1. General Characteristics of the Study Subjects

The general characteristics of the study subjects are shown in Table 1. A total of 57,303 students were included, including 29,841 male students and 27,462 female students. Regarding the school types, 65.9% were coeducational schools, 17.2% were all-male schools, and 16.9% were all-female schools. Metropolises accounted for 42.5% of the residences, and cities and rural areas accounted for 51.9%.

### 3.2. Whether Toothbrushing and Handwashing Practices after Lunch Were Associated with General Characteristics

Table 2 shows the toothbrushing and handwashing practices stratified by the general characteristics of the subjects. After lunch, 69.2% of female students brushed their teeth, which was statistically significantly higher than that of male students who brushed their teeth, which was 52.5% (*p* < 0.001). Moreover, 70.0% of female school students brushed their teeth after lunch, which was significantly higher than that of coeducational (60.2%) and male schools (52.5%) (*p* < 0.001). In the result of brushing behavior according to grade, high-school students showed a higher rate of toothbrushing than middle-school students, and significant differences were found at each grade level (*p* < 0.001). A total of 54.8% of students in metropolises, 63.2% of students in cities, and 79.1% of students in rural areas brushed their teeth after lunch, and the highest rate of practice was in rural areas. Regarding practicing handwashing before eating at school, 87.2% of the male students practiced it, which was statistically significantly higher than that of female students, 80.7% (*p* < 0.001). Moreover, 86.2% of male school students washed their hands before lunch, which was significantly higher than that of coeducational (84.9%) and female schools (78.6%) (*p* < 0.001).

Middle-school students showed a higher rate of handwashing than high-school students, and significant differences were found at each grade level (*p* < 0.001). In the classification according to the type of residence, the rural area showed the highest handwashing practice rate at 86.2%, but no significant difference was observed.

### 3.3. Association between Toothbrushing and Handwashing

Table 3 shows the results of the analysis of the association between toothbrushing and handwashing practices. Adolescents who practiced toothbrushing after lunch were 1.48 times more likely to practice handwashing before meals at school than those who did not practice toothbrushing after lunch (*p* < 0.001). In addition, an analysis of the association between toothbrushing and washing hands, after adjusting for gender, grade, school type, and residence, showed that adolescents who practiced toothbrushing after lunch were 1.87 times more likely to practice handwashing before meals at school than were those who did not (*p* < 0.001).

### 3.4. Association between Toothbrushing and Handwashing Practice in Relation to Personal Hygiene Education

The association between toothbrushing and handwashing practice is shown in Table 4. An analysis of students who participated in personal hygiene education at school within the last 12 months showed that adolescents who brushed their teeth after lunch were 1.66 times more likely to wash their hands before lunch at school than adolescents who did not brush their teeth after lunch (*p* < 0.001). In addition, an analysis of the association between toothbrushing and handwashing practices, after adjusting for gender, grade, school type, and residence, showed that adolescents who practiced toothbrushing after lunch were 1.98 times more likely to practice handwashing before lunch at school than those who did not brush their teeth after lunch (*p* < 0.001). An analysis of adolescents who did not participate in personal hygiene education at school showed that adolescents who practiced toothbrushing after lunch were 1.45 times more likely to practice handwashing before meals at school within the last 12 months (*p* < 0.001). Furthermore, an analysis of the association between toothbrushing and handwashing practices, after adjusting for gender, grade, school type, and residence, showed that adolescents who brushed their teeth after lunch were 1.80 times more likely to practice handwashing before lunch at school than those who did not brush their teeth after lunch (*p* < 0.001).

## 4. Discussion

In order to maintain and promote lifelong health, it is very important to have good health beliefs and habits during adolescence. Our hypothesis was that adolescents with good health habits have good hygiene behaviors, and that these behaviors are related to each other. Therefore, in our study, we investigated the relationship between handwashing and toothbrushing, which are representative hygiene behaviors.

In this study, according to the analysis of the practice of washing hands before lunch according to gender, male students showed significantly higher practice rates than female students. This finding is inconsistent with previous research results, with the reported gender-based handwashing practice rate in three South Pacific island countries being significantly higher for girls than boys [12]. These results were also found in the Health Behavior in School-Aged Children (HBSC) study and other prior studies conducted in Europe [13,14]. These results can be explained in part by the implementation of health education programs for women and adolescents classified as underprivileged in communities in the countries of concern, but additional studies on the association between gender and personal hygiene are expected to be needed in the future, considering the national health policies. In addition, this study showed that the rate of handwashing was lower among high-school students than among middle-school students, and a prior study showed that, as the students’ grade in high school increased, the number of students who said they did not wash their hands before lunch at school significantly increased. As a result, more careful monitoring and ongoing research on demographic and sociological factors affecting the practice of personal hygiene in schools are required [1]. Good hand hygiene can prevent the occurrence of various diseases. In a randomized controlled study that evaluated the effects of handwashing using soap on acute respiratory tract infection, agro-vacuum, and diarrhea prevention, the incidence of pneumonia in the handwashing group was 50% lower than that of the control group and 34% lower than that of the handwashing intervention group; moreover, handwashing with soap led to a 53% lower incidence of diarrhea [15]. It has also been reported that handwashing serves to reduce the spread of infectious diseases such as influenza, parasites, trachoma, neonatal infections, HIV-related infections, and enteritis [16,17,18,19,20]. Therefore, studies on how to habituate handwashing, which is most effective in reducing disease burden, during adolescence need to be conducted.

Insufficient oral hygiene-related behaviors in adolescence, such as an insufficient frequency of toothbrushing, increase the incidence of dental caries and, as a result, adversely affect not only the quality of life of adolescents but also the health of adults [21,22]. The practice of toothbrushing after lunch was analyzed in this study; 69.2% of female students and 52.5% of male students were found to perform this behavior, and the difference between groups was statistically significant. Similar results were found in previous studies. In a study on the toothbrushing practice of 231 Korean middle- and high-school students that was conducted using the 2017 Korean National Health and Nutrition Survey data, male students brushed their teeth 2.43 times per day and female students brushed them 2.64 times per day [23]. In addition, the rate of high-school students who brushed their teeth after lunch was significantly higher than that of middle-school students who brushed their teeth after lunch, and significant differences were identified across grade levels, which is consistent with the results in previous studies [24]. Behaviors related to oral health in adolescence can easily become habits, and it is a very important period in which behavioral changes in oral health are possible because it is the transition period from childhood to adulthood. Therefore, it is considered necessary to develop a systematic oral health education program along with a multifaceted analysis of factors affecting oral health behaviors in adolescence.

The results of this study showed that adolescents who brush their teeth after lunch are significantly more likely to practice handwashing before lunch at school than those who do not brush their teeth after lunch, and an association between oral hygiene and personal hygiene was confirmed. These results are consistent with those in previous studies, where 50.1% of students who brushed their teeth more than once a day always washed their hands after visiting the restroom, indicating that toothbrushing is closely related to personal hygiene habits [25]. In addition, in a previous study conducted in middle-school students from 15 Latin American countries, students who rarely brushed their teeth were 6.7 times more likely to not wash their hands than were those who brushed their teeth more often, and the results of an analysis of these associations by gender also confirmed a significant association [26].

The positive association between toothbrushing and handwashing was found to be stronger in adolescents who participated in health education. These results are consistent with those in prior research, which reported a 28.2% improvement in handwashing behavior and a 7.2% improvement in toothbrushing twice a day after hygiene education programs were implemented to improve knowledge and attitudes about handwashing and toothbrushing [27]. These results suggest that health promotion programs are needed to improve the hygiene-related behaviors of adolescents. The education, knowledge, and attitude of adolescent students regarding health hygiene are thought to have a large impact on hygiene practice, as well as on the students’ family and community. However, even if a school implements a health education program for personal hygiene management, it is difficult for students to perform personal hygiene management if the environment for toothbrushing and handwashing is poor; thus, the environment should be maintained, and a systematic health education program suitable for students should be developed and implemented.

However, this study had some limitations. First, this study was evaluated only for its association with cross-sectional studies. Secondly, the method used in this study was a questionnaire survey, and it is difficult to determine whether they answered correctly. In future studies, research and analysis to complement these points should be carried out. Notwithstanding these limitations, our study had some strengths. To the best of our knowledge, this is the first study to investigate oral hygiene practice and personal hygiene such as handwashing behavior according to health education experience among Korean adolescents using national representative standardized data. Secondly, the results of this study support the hypothesis that oral hygiene practices are related to personal hygiene.

Schools provide an important environment for the promotion of health at the most influential stage of individuals’ lives, during which individuals should develop sustainable health behaviors and maintain them throughout their entire lives [28]. Therefore, the development and implementation of comprehensive and systematic school-based oral health promotion programs is necessary. According to these findings, it is believed that comprehensive and systematic health policies are needed to improve youth hygiene and health.

## 5. Conclusions

Oral hygiene practices were found to be related to personal hygiene, as assessed by handwashing, in Korean adolescents. Additional studies are needed to develop ways of improving the hygiene and health of adolescents.

## Figures and Tables

**Table 1:** General characteristics of the study subjects (*n* = 57,303).

Variable		*N*	%
Gender	Boys	29,841	52.0
	Girls	27,462	48.0
School type	Coeducation	37,935	65.9
	All-boys schools	9818	17.2
	All-girls schools	9550	16.9
Grade	Middle school, 1st grade	9738	15.9
	Middle school, 2nd grade	9665	15.3
	Middle school, 3rd grade	9981	16.6
	High school, 1st grade	9273	17.1
	High school, 2nd grade	9044	16.5
	High school, 3rd grade	9602	18.5
Residence type	Metropolis	25,335	42.5
	City	27,471	51.9
	Rural area	4497	5.6

Values are presented as weighted percentages.

**Table 2:** Toothbrushing and handwashing practice data stratified by the general characteristics of the subjects (*n* = 57,303).

Variable		The Practice of Toothbrushing after a Meal			The Practice of Handwashing before a Meal		
		Yes	No	*p*-Value	Yes	No	*p*-Value
Gender							
	Boys	16,034 (52.5)	13,807 (47.5)	<0.001	26,143 (87.2)	3698 (12.8)	<0.001
	Girls	19,251 (69.2)	8211 (30.8)		22,274 (80.7)	5188 (19.3)	
School type							
	Coeducation	23,590 (60.2)	14,345 (39.8)	<0.001	32,353 (84.9)	5582 (15.1)	<0.001
	Boys’	5062 (52.5)	4756 (47.5)		8527 (86.2)	1291 (13.8)	
	Girls’	6633 (70.0)	2917 (30.0)		7537 (78.6)	2013 (21.4)	
Grade							
	Middle school 1st	5271 (51.8)	4467 (48.2)	<0.001	8823 (90.1)	915 (9.9)	<0.001
	Middle school 2nd	4996 (49.5)	4669 (50.5)		8346 (85.7)	1319 (14.3)	
	Middle school 3rd	5293 (50.9)	4688 (49.1)		8397 (83.7)	1584 (16.3)	
	High school 1st	6101 (63.9)	3172 (36.1)		7690 (82.9)	1583 (17.1)	
	High school 2nd	6399 (69.5)	2645 (30.5)		7469 (82,7)	1575 (17.3)	
	High school 3rd	7225 (74.7)	2377 (25.3)		7692 (80.1)	1910 (19.9)	
Residence type							
	Metropolis	14,210 (54.8)	11,125 (45.2)	<0.001	21,384 (84.1)	3951 (15.9)	0.115
	City	17,505 (63.2)	9966 (36.8)		23,110 (83.8)	4361 (16.2)	
	Rural area	3570 (79.1)	927 (20.9)		3923 (86.2)	574 (13.8)	

Values are presented as weighted percentages. The *p*-values were obtained from the complex samples crosstabs panel in SPSS.

**Table 3:** Relationship between toothbrushing and handwashing.

Independent Variable	Dependent Variable	Model 1		Model 2	
		Crude OR	95% CI	Adjusted OR *	95% CI
Toothbrushing practice	Handwashing practice (ref. no)	1.48	1.40–1.57	1.87	1.77–1.98

OR: odds ratio, CI: confidence interval. * Adjusted for gender, school type, grade, and residence type.

**Table 4:** Relationship between toothbrushing and handwashing stratified by students’ health education experience in school.

Independent Variable	Dependent Variable	Health Education Experience in School							
		Yes				No			
		Model 1		Model 2		Model 1		Model 2	
		Crude OR	95% CI	Adjusted OR *	95% CI	Crude OR	95% CI	Adjusted OR *	95% CI
Toothbrushing practice	Handwashing practice (ref. no)	1.66	1.51–1.82	1.98	1.80–2.19	1.45	1.35–1.54	1.8	1.68–1.92

OR: odds ratio, CI: confidence interval. * Adjusted for gender, school type, grade, and residence type.

## Data Availability

The datasets analyzed during the current study are available from the website of KYRBWS (http://www.kdca.go.kr/yhs/ (accessed on 10 January 2020)).

## References

[1] World Health Organization (2020). Water, sanitation, hygiene and waste management for the COVID-19 virus. World Health Organization Technical Brief.

[2] Curtis V., Cairncross S. (2003). Effect of washing hands with soap on diarrhrea risk in the community; A systematic review. Lancet Infect. Dis..

[3] Choi Y.S. (2014). Behaviors of hand washing practice Korean adolescents, 2011–2013: The Korea Youth Risk Behavior Web-based Survey. J. Korea Acad. Ind. Cooper. Soc..

[4] Korea Youth Risk Behavior Survey. https://www.kdca.go.kr/yhs/yhshmpg/main.do#.

[5] Park J.H., Lee M.J., Goo H.J. (2013). Oral health-related quality of life according to oral health behavior and awareness of middle school students in some regions. J. Korea Soc. Dent. Hyg..

[6] Addy M., Hunter M.L., Kingdon A., Dummer P.M., Shaw W.C. (1994). An 8-year study of changes in oral hygiene and periodontal health during adolescence. Int. J. Paediatr. Dent..

[7] David J., Wang N.J., Astrøm A.N., Kuriakose S. (2005). Dental caries and associated factors in 12-year-old schoolchildren in Thiruvananthapuram, Kerala, India. Int. J. Paediatr. Dent..

[8] Park J.H., Kim C.S. (2015). Relationship between health behavior and oral symptoms in Korean adolescents. J. Korean Soc. Dent. Hyg..

[9] Oh J.S., Lee S.H. (2019). A Convergence Study on the Relationship of Body-Shape Perception and Periodontal Diseases in Female Adolescents. J. Korea Converg. Soc..

[10] Korea Youth Risk Behavior Survey. https://www.kdca.go.kr/yhs/yhshmpg/result/mainStatsGraph.do.

[11] The Ministry for Health and Welfare of the Republic of Korea (2020). Korea Centers for Disease Control and Prevention. Korea Youth Risk Behavior Survey Raw Data Use Guidelines.

[12] Tran D., Phongsavan P., Bauman A.E., Havea D., Galea G. (2006). Hygiene Behaviour of Adolescents in the Pacific: Associations with Socio-demographic, Health behaviour and School Environment. Asia Pac. J. Public Health.

[13] Taani D.S., al-Wahadni A.M., al-Omari M. (2003). The effect of frequency of toothbrushing on oral health of 14–16 year olds. J. Irish Dent. Assoc..

[14] Borghi J., Guinness L., Ouedraogo J., Curtis V. (2020). Is hygiene promotion cost effective? A case study in Burkina Faso. Trop. Med. Int. Health.

[15] Luby S.P., Agboatwalla M., Feikin D.R., Painter J., Billhimer W., Altaf A., Hoekstra R.M. (2005). Effect of handwashing on child health: A randomised controlled trial. Lancet.

[16] Ejere H.O.D., Alhassan M.B., Rabiu M. (2015). Face washing promotion for preventing active trachoma. Cochrane. Syst. Rev..

[17] Blencowe H., Cousens S., Mullany L.C., Lee A.C.C., Kerber K., Wall S., Darmstadt G.L., Lawn J.E. (2011). Clean birth and postnatal care practices to reduce neonatal deaths from sepsis and tetanus: A systematic review and Delphi estimation of mortality effect. BMC. Public Health.

[18] Curtis V., Schmidt W., Luby S., Florez R., Touré O., Biran A. (2011). Hygiene: New hopes, new horizons. Lancet. Infect. Dis..

[19] Greenland K., Cairncross S., Cumming O., Curtis V. (2013). Can we afford to overlook hand hygiene again?. Trop. Med. Int. Health.

[20] Freeman M.C., Clasen T., Brooker S.J., Akoko D.O., Rheingans R. (2013). The impact of a school-based hygiene, water quality and sanitation intervention on soil-transmitted helminth reinfection: A cluster-randomized trial. Am. J. Trop. Med. Hyg..

[21] Petersen P.E., Bourgeois D., Ogawa H., Estupinan-Day S., Ndiaye C. (2005). The global burden of oral diseases and risks to oral health. Bull. World. Health Organ..

[22] Zaborskis A., Milciuviene S., Narbutaite J., Bendoraitiene E., Kavaliauskiene A. (2010). Caries experience and oral health behavior among 11–13-year-olds: An ecological study of data from 27 European countries, Israel, Canada and USA. Commun. Dent. Health.

[23] Park H.J. (2020). Factors that Influences Daily Toothbrushing Frequency and Use of Oral Health Care Products for Adolescents. J. KoCon. A.

[24] Hwang M.J., Seong J.M., Kim J.H., Yoo S.M., Park Y.D. (2009). The relationship between oral health behaviors and sociodemographic characteristics in Korean adolescents. J. Korean Acad. Dent. Health.

[25] Nzioka B.M., Nyaga J.K., Wagaiyu E.G. (1993). The relationship between toothbrushing frequency and personal hygiene habits in Teenagers. East. Afr. Med. J..

[26] McKittrick T.R., Jacobsen K.H. (2015). Oral Hygiene and Handwashing Practices among Middle School Students in 15 Latin American and Caribbean Countries. West. Indian Med. J..

[27] Umair Q., Anwar S. (2019). Hand washing behavior change effect of community-based hygiene and sanitation intervention in low resource setting. J. Public Health.

[28] Kwan S.Y., Petersen P.E., Pine C.M., Borutta A. (2005). Health-promoting schools: An opportunity for oral health promotion. Bull. World. Health Organ..

